# Characteristics and Prognosis of Acquired Resistance to Immune Checkpoint Inhibitors in Gastrointestinal Cancer

**DOI:** 10.1001/jamanetworkopen.2022.4637

**Published:** 2022-03-29

**Authors:** Na Zhuo, Chang Liu, Qi Zhang, Jian Li, Xiaotian Zhang, Jifang Gong, Ming Lu, Zhi Peng, Jun Zhou, Xicheng Wang, Xi Jiao, Yujiao Wang, Yanni Wang, Mengting Gao, Lin Shen, Zhihao Lu

**Affiliations:** 1 Department of Gastrointestinal Oncology, Key Laboratory of Carcinogenesis and Translational Research (Ministry of Education), Peking University Cancer Hospital & Institute, Beijing, People’s Republic of China

## Abstract

**Importance:**

With the expanding use of immune checkpoint inhibitors (ICIs) in gastrointestinal (GI) cancer, the occurrence of acquired resistance (AR) has gradually emerged. However, the progression patterns and survival of patients with AR to ICIs are still unknown.

**Objective:**

To explore the characteristics and prognosis of AR after ICI therapy in patients with advanced GI cancer.

**Design, Setting, and Participants:**

This cohort study screened patients with advanced GI cancer treated with ICIs between January 14, 2016, and December 31, 2020, at Peking University Cancer Hospital. Initial response was defined as complete response, partial response, or stable disease longer than 6 months as assessed by Response Evaluation Criteria in Solid Tumors version 1.1. Progression was also based on Response Evaluation Criteria in Solid Tumors version 1.1. Progression or death after the initial response was defined as AR. Oligoprogression of AR was defined as 2 or more disease sites progression. The current status of AR in GI cancer and the patterns of AR and its prognosis were evaluated. The site of AR and subsequent management were also assessed. Data were analyzed from June to August 2021.

**Exposures:**

Patients in the cohort were treated with mono-ICI or combination therapy.

**Main Outcomes and Measures:**

Kaplan-Meier analyses and log-rank tests were conducted for overall survival analyses. Univariate and multivariate Cox analyses were conducted to determine the prognostic implications of each variable.

**Results:**

Of the 1124 patients who received ICIs, 373 (33.2%) patients (282 men [75.6%]; median [IQR] age, 62 [54-68] years) achieved an initial response, and 173 (46.4%) patients (137 men [79.2%]; median [IQR] age, 61 [54-67] years) developed AR. Almost all patients (167 patients [96.5%]) developed AR within 24.0 months. Progression patterns of AR were most commonly oligoprogression (122 patients [70.5%]) rather than polymetastatic progression (38 patients [22.0%]) and were associated with a good prognosis (38.5 vs 14.0 months; hazard ratio, 0.37; 95% CI, 0.18-0.74; *P* < .001). Lymph nodes (101 patients [58.4%]) appeared to be the most common site of AR. Management after AR was mainly systemic therapy (96 patients [55.5%]).

**Conclusions and Relevance:**

Oligoprogression was the most common pattern of AR progression, and lymph nodes were the most susceptible site for AR. Further study will be needed to determine the most favorable management for AR.

## Key Points

**Question:**

What are the characteristics and prognosis of acquired resistance to immune checkpoint inhibitors in gastrointestinal (GI) cancer?

**Findings:**

In this cohort study of 1124 patients with advanced GI cancer, a high frequency (46.4%) of acquired resistance (AR) was found. Oligoprogression was the most common pattern of AR progression, and lymph nodes were the most susceptible site for AR.

**Meaning:**

Given that large-scale retrospective analyses of patients with GI cancer treated with immune checkpoint inhibitors are lacking, this study will provide useful information and a deeper understanding to clinicians regarding AR in patients with advanced GI cancer.

## Teaser

This cohort study examines patients with gastrointestinal cancer to evaluate the characteristics of acquired resistance to immune checkpoint inhibitors.

## Introduction

Immune checkpoint inhibitors (ICIs) have become one of the most promising approaches in cancer immunotherapy.^1,2^ In the field of gastrointestinal (GI) cancer, ICIs have made great breakthroughs in advanced esophageal, gastric, and deficient mismatch repair (dMMR) colorectal cancers, showing encouraging efficacy and bringing new hope for patients with cancer.^3,4,5,6^

Although ICIs have shown great success in many types of solid tumors, most patients who initially respond to immunotherapy develop acquired resistance (AR) over time, thus limiting the durability of immunotherapy. In the evaluation of some clinical trials, the rate of AR varied among tumor types (eg, nearly 50% in non–small cell lung cancer [NSCLC] and approximately 35% in melanoma), especially in GI cancer, where the rate was as high as 42% to 71%.^7,8,9,10^ Therefore, a comprehensive understanding of the clinical features and prognosis of AR is essential for clinical work. Several studies have attempted to characterize the progression of AR to ICIs in other solid tumors on the basis of clinical data analysis. For example, a 2018 study by Gettinger et al^11^ identified and evaluated 26 patients with NSCLC who developed AR to ICI therapy. This study revealed that lymph nodes were the main progression site of AR and showed different follow-up management strategies for patients with AR. In another study on AR in NSCLC, Xu and colleagues^12^ found that the major progression pattern of AR was oligoprogression. They also concluded that local radiotherapy combined with continuous immunotherapy would benefit this group of patients. Similar findings have been reported in melanoma by Pires da Silva et al,^13^ who identified an association between the size and site of individual metastases and patterns of response, progression, and clinical outcome.

However, the clinical features and survival outcomes of AR to ICIs in patients with GI cancer are still unknown. Therefore, we performed a retrospective cohort study to comprehensively describe the clinical characteristics and prognosis of AR in advanced GI cancer.

## Methods

### Patient Collection and Response Assessment

Patients with advanced GI cancer receiving treatment with ICIs in the Department of GI Oncology, Peking University Cancer Hospital & Institute, between January 14, 2016, and December 31, 2020, were screened in this large retrospective study. This study was exempted by the institutional review board at the Peking University Cancer Hospital and was performed according to the Declaration of Helsinki.^14^ Written informed consent was given by each individual before the treatment. The study enrolled patients with esophageal cancer, gastric cancer or esophagogastric junction (EGJ) cancer, intestinal cancer (including colorectal and small intestinal cancers), hepatobiliary-pancreatic cancer, and neuroendocrine tumors. Patients were divided into 2 different ICI therapy cohorts. Mono-ICI was defined as programmed cell death protein 1/programmed cell death 1 ligand 1 (PD1/PD-L1) inhibitor or PD1/PD-L1 inhibitor combined with cytotoxic T-lymphocyte–associated protein 4 inhibitor or transforming growth factor–β inhibitor therapy. Combination therapy was defined as ICI therapy combined with chemotherapy and/or targeted therapy.

Initial response was considered achieving complete response (CR), partial response (PR), or stable disease more than 6 months after ICI therapy. Progression was defined as an increase in the size of the primary tumor and or the appearance of a new lesion. AR was defined as progression or death after initial response. Imaging scans of individual patients during ICI therapy were reviewed by trained radiologists, and responses were determined according to Response Evaluation Criteria in Solid Tumors version 1.1. Patient progression sites were captured, and follow-up management after AR was recorded.

### Predefined Progression Patterns of AR

The pattern of progression was determined using predefined characteristics at the time that any tumor site met the criteria for the progression of disease, whichever occurred first. Oligoprogression and polymetastatic progression of AR were defined as progression in less than 2 or more than 3 disease sites, respectively. Original progression was defined as the progression of lesions which were originally involved before immunotherapy.

### Statistical Analysis

Progression-free survival (PFS) was defined as the time from the first dose of ICIs to radiological disease progression or death from any cause. Overall survival (OS) was defined as the time from the first dose of ICIs to death from any cause. The time to AR was defined as the time from the first dose of ICIs to AR. OS was estimated using a Kaplan-Meier survival analysis and 2-sided log-rank test. The association of each variable with survival of patients with AR was completed by univariate and multivariate Cox regression analysis. *P* < .05 was considered to be significant. Statistical analyses were performed with GraphPad Prism statistical software version 8.0 (GraphPad Software) and R statistical software version 3.6.2 (R Project for Statistical Computing).

## Results

### Patients

**Figure 1.**
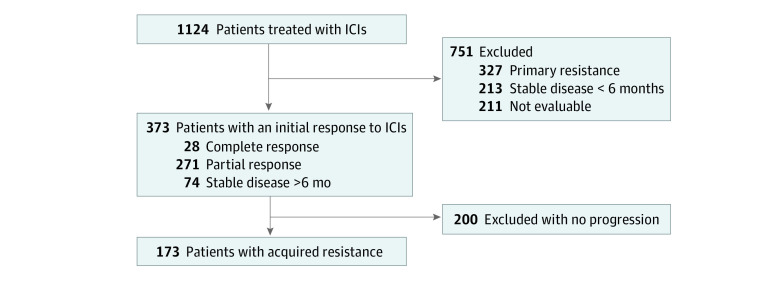
Patient Enrollment Flowchart Initial response was considered achieving complete response, partial response, or stable disease more than 6 months after initiation of treatment with immune checkpoint inhibitors (ICIs) in patients evaluated by Response Evaluation Criteria in Solid Tumors version 1.1. Acquired resistance was defined as disease progression in patients who achieved an initial response to ICIs.

All 1124 patients with GI cancer who received ICIs at Peking University Cancer Hospital & Institute between January 14, 2016, and December 31, 2020, were included in the total cohort (Figure 1). In addition to 211 patients whose outcomes were not evaluable, 327 patients with primary resistance and 213 patients with stable disease longer than 6 months were also excluded. Of 1124 patients, 373 (33.2%) with an initial response were identified, 299 patients (26.6%) achieved a CR or PR, and 74 patients (6.6%) achieved stable disease longer than 6 months. The cohort of 373 included 282 men (75.6%; median [IQR] age, 62 [54-68] years). All patients in the cohort of 373 had pathologically or cytologically confirmed primary GI cancer, except for a few neuroendocrine tumors of unknown origin (4 patients [1.0%]) that were diagnosed according to the clinicopathological features. The median OS for patients with an initial response was not reached (95% CI, 14.6 to not reached) (eFigure 1 in the Supplement). The median follow-up time was 15.4 months (95% CI, 14.6 to 16.6 months).

Of the 373 patients, a total of 173 patients (46.4%) were included in the AR cohort after the initial response, most of whom were patients with CR or PR whose disease had progressed (145 patients [83.8%]). A total of 28 patients (16.2%) progressed from stable disease more than 6 months. The median OS for patients with AR was 20.9 months (95% CI, 14.6-27.1 months) (eFigure 1 in the Supplement). Demographic and baseline clinical characteristics are shown in the Table.

**Table.** Demographic and Baseline Characteristics of Patients With an Initial Response

Characteristic	Patients, No. (%)	
	Response (n = 373)	Acquired resistance (n = 173)
Age, median (IQR), y	62 (54-68)	61 (54-67)
Sex		
Male	282 (75.6)	137 (79.2)
Female	91 (24.4)	36 (20.8)
Eastern Cooperative Oncology Group Performance Status score		
0-1	354 (94.9)	156 (90.2)
2	14 (3.8)	12 (6.9)
3	5 (1.3)	5 (2.9)
Body mass index, median (IQR)^a^	22.2(20.06-24.52)	22.1 (19.79-24.32)
Smoking history		
No	192 (51.5)	81 (46.8)
Yes	181 (48.5)	96 (53.2)
Alcohol consumption		
No	214 (57.4)	90 (50.0)
Yes	159 (42.6)	83 (48.0)
Primary tumor site		
Esophageal	82 (22.0)	40 (23.1)
Gastric or esophagogastric junction	182 (48.8)	92 (53.2)
Colorectal	63 (16.9)	16 (9.2)
Small intestinal	5 (1.3)	2 (1.2)
Pancreatic	9 (2.4)	7 (4.0)
Hepatobiliary	28 (7.5)	14 (8.1)
Histological profile		
Adenocarcinoma	263 (70.5)	119 (68.8)
Squamous cell	82 (22.0)	39 (22.5)
Neuroendocrine	16 (4.3)	10 (5.8)
Histological grade		
Well or moderately differentiated	198 (53.1)	91 (52.6)
Poorly differentiated	142 (38.1)	61 (35.2)
Unknown	33 (8.8)	21 (12.1)
Site of metastases or recurrence		
Lymph nodes	320 (85.8)	155 (89.6)
Liver	122 (32.7)	76 (43.9)
Lung	65 (17.4)	30 (17.3)
Bone	28 (7.5)	13 (7.5)
Spleen	15 (4.0)	7 (4.0)
Adrenal gland	16 (4.3)	13 (7.5)
Brain	4 (1.1)	2 (1.2)
Ovary	7 (1.9)	3 (1.7)
Peritoneum	58 (15.5)	36 (20.8)
Anastomosis	19 (5.1)	10 (5.8)
Previous therapy		
Surgery	85 (22.8)	38 (22.0)
Radiotherapy	81 (21.7)	41 (23.7)
Previous systemic therapies, No.		
1	199 (53.4)	86 (49.7)
2	105 (28.2)	46 (26.6)
>2	69 (18.5)	41 (23.7)
Regimen		
Immune checkpoint inhibitors	152 (40.8)	68 (39.3)
Plus chemotherapy	130 (34.9)	69 (39.9)
Plus targeted therapy	64 (17.2)	23 (13.3)
Plus chemotherapy and targeted therapy	27 (7.2)	13 (7.5)
Response		
Complete response	28 (7.5)	2 (1.2)
Partial response	271 (72.7)	143 (82.7)
Stable disease >6 mo	74 (19.8)	28 (16.2)
MSI status		
MSI-H/dMMR	110 (29.5)	32 (18.5)
MSS/pMMR	181 (48.5)	100 (57.8)
Not applicable^b^	82 (22.0)	41 (23.7)

Abbreviations: MSI-H/dMMR, microsatellite instability-high/deficient mismatch repair; MSS/pMMR, microsatellite stable/proficient mismatch repair.

^a^ Body mass index is calculated as weight in kilograms divided by height in meters squared.

^b^ MSI status testing is not routinely tested in all gastrointestinal cancer (eg, esophageal cancer, hepatobiliary cancer, and so forth), and MSI status data are not avaliable in these cancer patients.

### Characteristics of AR

**Figure 2.**
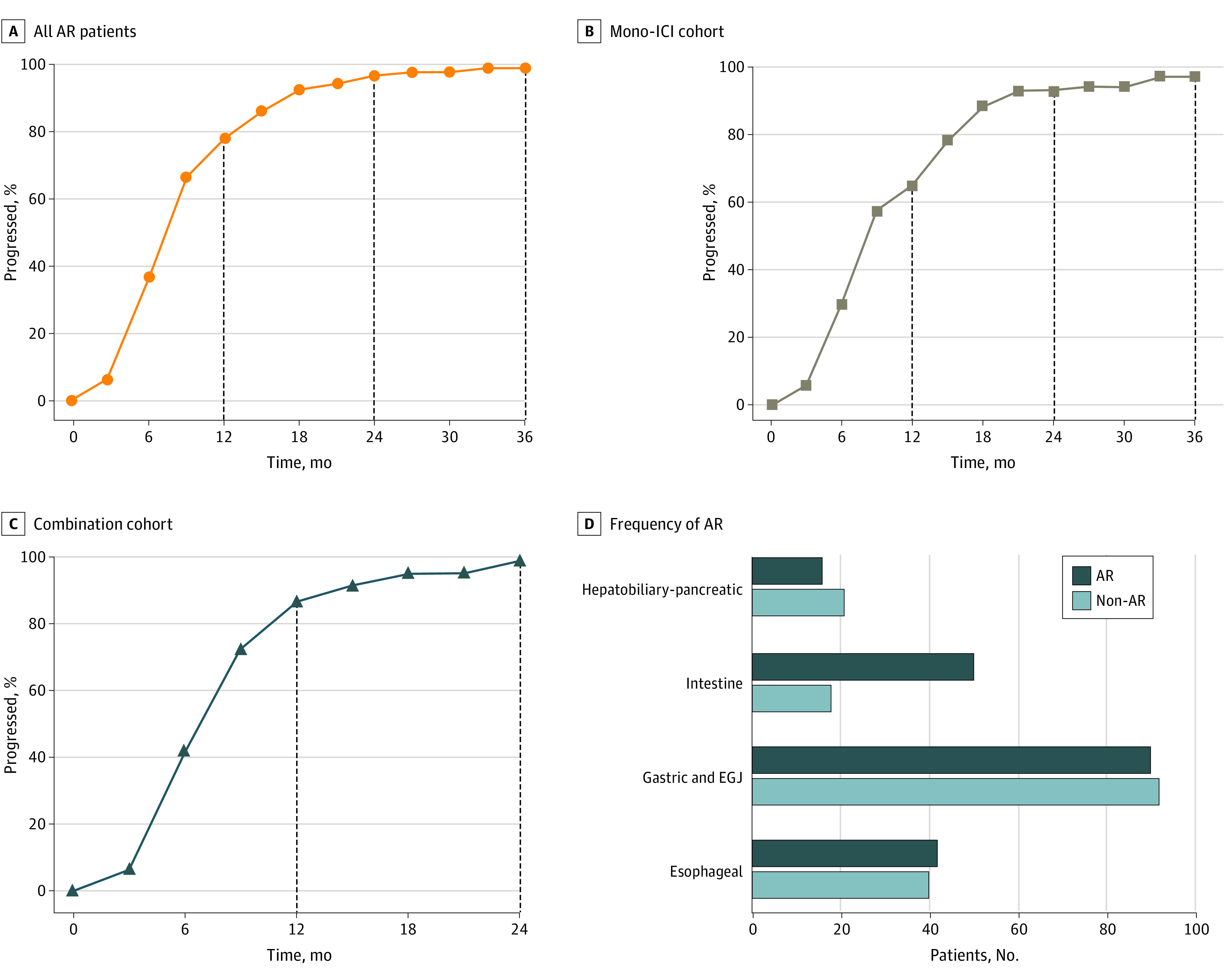
Time to Acquired Resistance (AR) After the Initial Response Panel A shows the rate of AR over time in all patients. Panels B and C show the rate of AR over time in the immune checkpoint inhibitor (ICI) monotherapy cohort or the combination cohort. Panel D shows the frequency of AR in patients with gastrointestinal (GI) cancer different primary foci. EGJ indicates esophagogastric junction.

In our AR cohort (173 patients), the median time to AR was 7.3 months (95% CI, 6.4-8.2 months) and almost all patients (167 patients [96.5%]) developed AR within 2 years (Figure 2A). The 1-year and 2-year survival rates for patients with AR were 78.7% (95% CI, 72.1%-85.2%) and 48.4% (95% CI, 38.5%-57.5%), respectively. We next evaluated the time to AR in patients with different ICI therapies. A total of 68 patients (39.3%) were treated with mono-ICI therapy, and 105 patients (60.7%) were treated with combination therapy (Table). In the mono-ICI therapy cohort, the median OS for patients with AR was 22.9 months (95% CI, 14.7-31.2 months), and the median time to AR was 8.1 months (95% CI, 7.2-9.0 months). A total of 44 patients (64.7%) experienced AR within 12.0 months. Nearly all patients (63 patients [92.6%]) developed AR within 2 years (Figure 2B). In the combination therapy cohort, the median OS was 26.1 months (95% CI, 12.5-40.0 months), and the median time to AR was 6.8 months (95% CI, 5.8-7.7 months). A total of 91 patients (86.7%) developed AR within 12.0 months in the combination therapy cohort, and 104 patients (99.0%) developed AR within 24.0 months (Figure 2C).

We also characterized the AR in different primary foci. The frequency of AR differed among patients with GI cancer (Figure 2D). Among patients with esophageal cancer who responded to ICIs, 40 patients (48.8%) developed AR. The median OS was 15.6 months (95% CI, 11.6-16.4 months), and the median time to AR was 7.8 months (95% CI, 7.6-11.6 months). AR was observed in 38 patients (95.0%) within 24.0 months (Figure 2D; eFigure 2 in the Supplement). Patients with gastric cancer or EGJ cancer accounted for nearly half of all patients with an initial response, and 92 patients (50.5%) achieved AR. For these patients, the median OS was 19.6 months (95% CI, 10.6-28.6 months), and the median time to AR was 6.8 months (95% CI, 5.5-8.0 months). A total of 90 patients (97.8%) progressed within 2 years (Figure 2D; eFigure 2 in the Supplement). Among patients with intestinal cancer 61 of 68 patients had microsatellite instability-high/dMMR tumors, including 63 patients with colorectal cancer and 5 patients with small intestine cancer; the frequency of AR was only 26.5%, which was lower than the frequencies of AR in patients with other GI cancers. The median OS was not reached, and the median time to AR was 8.1 months (95% CI, 7.1-11.4 months). These patients achieved AR within 2 years (Figure 2D; eFigure 2 in the Supplement). For hepatobiliary and pancreatic cancers, AR occurred in more than half of the patients (21 patients [56.8%]). The median OS for these patients was also not reached, and the median time to AR was 7.6 months (95% CI, 6.1-14.9 months). The rate of AR in this group of patients within 2 years was 90.5% (19 patients) (Figure 2D; eFigure 2 in the Supplement).

### Progression Patterns of AR

We next analyzed the patterns of progression in patients with AR and the association with clinical outcomes. Patterns of progression were predefined to assess the heterogeneity among them.

**Figure 3.**
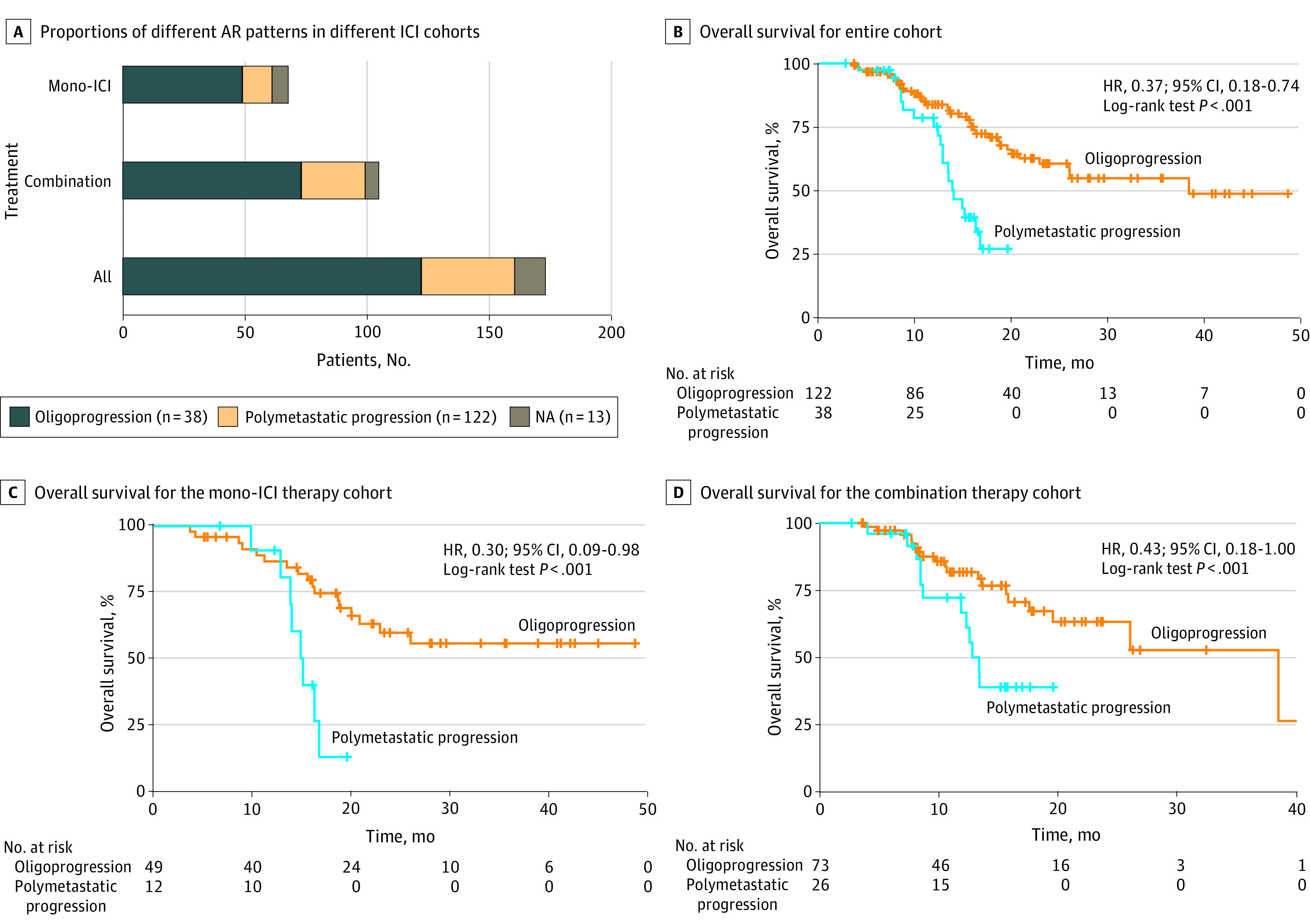
Progression Patterns of Acquired Resistance (AR) Panel A shows proportions of different AR patterns in different immune checkpoint inhibitor (ICI) cohorts. Panel B shows Kaplan-Meier overall survival analysis curves with different progression patterns in patients with AR. Panel C shows overall survival of patients with different progression patterns in the mono-ICI therapy cohort. Panel D shows overall survival with different progression patterns in the combination therapy cohort. HR indicates hazard ratio; NA, not available.

Oligoprogression was more common than polymetastatic progression in the AR cohort, occurring in 122 patients (70.5%). Polymetastatic progression occurred in 38 patients (22.0%), and 13 patients died without radiographic progression. The proportions of oligoprogression and polymetastatic progression were similar in immunotherapy cohorts. In the mono-ICI cohort, oligoprogression and polymetastatic progression occurred in 49 patients (72.1%) and 12 patients (17.6%), respectively. In the combination cohort, oligoprogression and polymetastatic progression occurred in 73 patients (69.5%) and 26 patients (24.8%), respectively (Figure 3A).

We next compared the clinical outcomes of patients with different AR patterns in GI cancer. We found that patients with oligoprogression had improved survival compared with those with polymetastatic progression (38.5 vs 14.0 months; hazard ratio [HR], 0.37; 95% CI, 0.18-0.74; *P* < .001) (Figure 3B). In the patients with oligoprogression, the 1-year and 2-year survival rates were 72.4% (95% CI, 63.1%-79.8%) and 29.8% (95% CI, 20.4%-39.8%), respectively; the 1-year survival rate for patients with systemic progression was 75.1% (95% CI, 56.3%-86.8%). More specifically, oligoprogression was associated with better clinical outcomes compared with polymetastatic progression in both the mono-ICI cohort (unreached vs 15.1 months; HR, 0.30; 95% CI, 0.09-0.98; *P* < .001) and the combination therapy cohort (38.5 vs 12.9 months; HR, 0.43; 95% CI, 0.18-1.00; *P* = .02) (Figure 3C and 3D).

We also conducted univariate and multivariate Cox analyses for variables associated with the outcome of patients with AR. For the univariate analysis, the results showed that only progression patterns were significantly associated with the OS of patients with AR (HR, 0.34; 95% CI, 0.19-0.60; *P* < .001) (eTable 1 in the Supplement). Then, we selected progression patterns and other clinically significant univariate variables for multivariate analysis. We found that oligoprogression was independently associated with survival in patients with AR who also have GI cancer (HR, 0.33; 95% CI, 0.17-0.66; *P* = .002) (eFigure 3 in the Supplement).

### Site of AR

**Figure 4.**
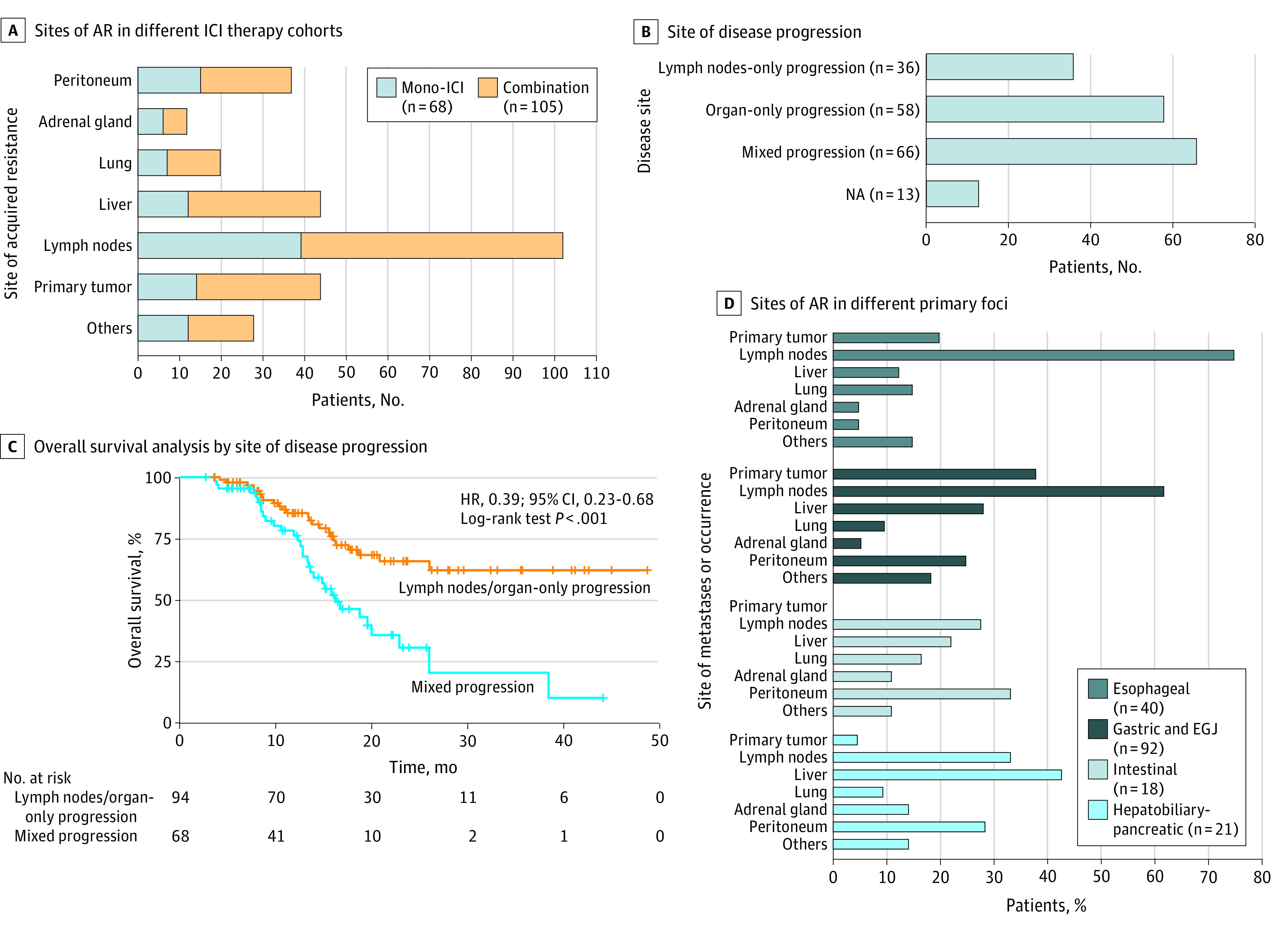
Sites of Acquired Resistance (AR) Panel A shows the sites of AR in different immune checkpoint inhibitor (ICI) therapy cohorts. Panel B shows patients with AR were divided according to the progression of disease in lymph nodes and/or organs. Panel C shows Kaplan-Meier overall survival analysis curves for patients with AR according to lymph nodes and/or organ progression. Panel D shows sites of AR in different primary foci; no patients with intestinal cancer had AR in the primary tumor site. EGJ indicates esophagogastric junction; HR, hazard ratio; NA, not available.

We counted all sites of progression in patients with AR, including primary foci, lymph nodes, and peritoneum. Lymph nodes were included from multiple sites, such as the mediastinum, axilla, abdomin, and pelvis. More than half of the patients (103 patients [59.5%]) showed progression to the original lesion (eTable 2 in the Supplement) and a total of 19 patients (11.0%) developed new lesions. When considering specific progression sites, AR commonly occurred in lymph nodes (101 patients [58.4%]), followed by the liver (44 patients [25.4%]) and primary tumor (44 patients [25.4%]). In addition, lymph nodes were the most common sites of AR in both the mono-ICI (38 patients [55.9%]) and the combination therapy (63 patients [60.0%]) cohorts (Figure 4A).

We also classified the sites of AR as lymph node or organ-only progression or mixed progression (progression in both lymph nodes and organs). A total of 36 patients (20.8%) had limited lymph node progression, 58 patients (33.5%) had organ-only progression, and the largest proportion of patients (66 patients [38.2%]) had mixed progression. Patients with mixed progression had much lower OS than patients with lymph node or organ-only progression (16.3 months vs unreached; HR, 0.39; 95% CI, 0.23-0.68; *P* < .001) (Figure 4B and C).

We further compared the progression sites according to the different primary tumors. Lymph nodes remained the most predominant AR site in esophageal and gastric or EGJ cancer, with 30 patients (75.0%) and 57 patients (62.0%), respectively. The peritoneum (6 patients [33.3%]) was the main site of progression for intestinal cancer, whereas the main site of progression for hepatobiliary and pancreatic cancers was the liver (9 patients [42.9%]) (Figure 4D).

### Management After AR

Finally, we counted the treatment patterns of these patients with AR. A total of 149 patients (86.1%) had available follow-up treatment information (eTable 2 in the Supplement). Follow-up management can be categorized as best supportive care, local therapy, and systemic therapy. Of these patients, 30 patients (17.3%) were treated with best supportive care, 96 patients (55.5%) received systemic therapy, and 23 patients (13.3%) received local therapy.

Systemic therapy refers to chemotherapy, maintaining the original immunotherapy, phase 1 clinical trials, and targeted therapy. Most patients received chemotherapy (41 patients [23.7%]), followed by those who maintained the original immunotherapy (22 patients [12.7%]). Local therapy involved interventional therapy and radiotherapy; 16 patients (9.2%) and 7 patients (4.0%) received radiotherapy and interventional therapy, respectively. We tried to compare the associations of systemic vs local therapy with long-term survival benefits. The median OS for patients who received systemic therapy was not reached, and the median OS for patients who received local therapy was also not reached. There was no significant difference in OS between the 2 groups.

## Discussion

In this cohort study, we reviewed a large cohort of patients receiving immunotherapy and comprehensively described the frequency, progression patterns, and sites of AR in GI cancer. We found oligoprogression was the most common pattern of AR progression, and lymph nodes were the most susceptible site for AR.

A previous study^7^ estimated the rate of AR in many types of solid tumors. For example, the rates of AR were approximately 32% to 64% in NSCLC, 35% to 54% in head and neck squamous carcinoma, and 12% to 60% in melanoma.^7^ In GI cancer (mainly esophageal, gastric, and EGJ carcinoma), the rate of AR ranged from 41% to 71%. In the present study, we confirmed the high rate (46.4%) of AR in GI cancer, which was consistent with a previous study. Notably, the rate of AR in intestinal cancer (26.5%) appeared to be much lower than that in other GI cancers. This phenomenon may be associated with the fact that most of the patients with intestinal cancer (61 of 68) in our cohort had microsatellite instability-high/dMMR tumors. Furthermore, we also noticed that the median time to AR was shorter in GI cancer than in other cancers. In our cohort, the time to AR was approximately 7 to 8 months for either mono or combination ICIs or different primary sites, and most patients developed AR within 24.0 months.^11,13,15^ Accumulating evidence suggests that dynamic cross-talk between cancer cells and the immune microenvironment is associated with the occurrence of AR.^7^ Although the current mechanisms of AR are summarized as defects in tumor antigen presentation, neoantigen depletion, tumor-mediated immunosuppression or exclusion, and additional inhibitory checkpoints, the exploration of the mechanism is still far from sufficient.^16,17,18,19,20^ Much work remains to be done to provide patients with personalized response strategies and to improve prognosis with an increased understanding of the mechanism of AR.

The progression patterns of AR have been previously reported in melanoma and NSCLC but, to our knowledge, have not been explored in GI cancer.^12,15,21^ In the present study, we classified the progression patterns of AR into oligoprogression and polymetastatic progression on the basis of findings from previous studies. Consistent with studies on other solid tumors, we found that the pattern of AR was predominantly oligoprogression and was associated with a good prognosis. To distinguish the AR progression patterns of different ICI therapies, we also validated the definition and survival in mono-ICI and combination therapy cohorts. The results confirmed the reliability of this definition approach. Given the limited number of similar studies available, more clinical data are still needed to support these results.

A notable finding in this study was that a large proportion of patients with AR had lymph node progression; however, this was not observed for all GI cancers. For intestinal cancer (including colorectal and small intestinal cancers), the most common site of AR was the peritoneum, while the main site of AR for hepatobiliary-pancreatic cancer was the liver. This may be due to the inherent metastatic heterogeneity and metastatic preference of the cancer, as lymph nodes are one of the most common sites of metastasis in esophageal and gastric cancers, especially in advanced stages.^22,23,24,25^ Peritoneal metastasis is the most common form of metastasis for dMMR intestinal cancer, while hepatobiliary-pancreatic cancer is dominated by liver metastasis.^26,27,28^

At the end of the study, we tried to explore the treatment that would be most beneficial for the long-term survival of patients with AR. Previous limited studies^12,13,15^ have shown that in melanoma, different patterns of progression of AR were associated with OS with combination immunotherapy, while in patients with advanced NSCLC with AR, studies have suggested that local treatment and/or continuation of ICIs after AR may be an effective option. This revealed that there was also great variability in the management of different cancers after AR. In the present study, we found that systemic therapy was the predominant treatment for patients with AR in GI cancer, with chemotherapy being the mainstay. Considering the inclusion of multiple GI cancer species in our cohort and the inherent heterogeneity of GI cancer, it was difficult to conclusively identify the most favorable management for these patients. More clinical retrospective studies are still needed to further explore the most favorable management for patients with GI cancer who experience AR.

### Limitations

Our study has limitations. Because this was a retrospective study, the potential selection bias from a single center may affect the clinical application of our results. In addition, the treatments after AR were complicated and diverse, and we have not yet obtained the most beneficial treatment for these patients. More large-scale clinical studies are needed to further validate this definition and the reliability of the results.

## Conclusions

In summary, to our knowledge, this was the first retrospective study on the clinical characteristics and prognosis of AR in advanced GI cancer. Our findings showed a high prevalence of AR in GI cancer, oligoprogression was the predominant AR pattern with a good prognosis, and the most common sites of AR were concentrated in the lymph nodes. The most beneficial treatment for patients with AR needs further exploration. In particular, we performed a more detailed subgroup analysis for different ICI therapies and primary sites, aiming to provide more accurate information on the characteristics of AR in different patient populations. Our study may provide useful information and a deeper understanding to clinicians regarding AR in patients with advanced GI cancer.

## Notes

Supplement. **eFigure 1.** Overall Survival of Initial Response Patients and Acquired Resistance Patients **eFigure 2.** The Rate of Acquired Resistance Over Time in Different Primary Sites **eTable 1.** Univariate Analysis of Hazard Ratio For OS in AR Cohorts **eFigure 3.** Multivariate Analysis of Hazard Ratio for OS in AR Cohorts **eTable 2.** Characteristics of Patients With Acquired Resistance
