# Supplementary material for: Characteristics and Prognosis of Acquired Resistance to Immune Checkpoint Inhibitors in Gastrointestinal Cancer
Source: JAMA Netw Open. 2022 Mar 29;5(3):e224637. doi: 10.1001/jamanetworkopen.2022.4637 (PMC8965636; doi:10.1001/jamanetworkopen.2022.4637)
Supplement: Supplement. — eFigure 1. Overall Survival of Initial Response Patients and Acquired Resistance Patients eFigure 2. The Rate of Acquired Resistance Over Time in Different Primary Sites eTable 1. Univariate Analysis of Hazard Ratio For OS in AR Cohorts eFigure 3. Multivariate Analysis of Hazard Ratio for OS in AR Cohorts eTable 2. Characteristics of Patients With Acquired Resistance [file jamanetwopen-e224637-s001.pdf]

Supplemental Online Content

Zhuo N, Liu C, Zhang Q, et al. Characteristics and prognosis of acquired resistance to immune checkpoint inhibitors in gastrointestinal cancer. *JAMA Netw Open*. 2022;5(3):e224637.
doi:10.1001/jamanetworkopen.2022.4637

eFigure 1. Overall Survival of Initial Response Patients and Acquired Resistance Patients
eFigure 2. The Rate of Acquired Resistance Over Time in Different Primary Sites
eTable 1. Univariate Analysis of Hazard Ratio for OS in AR Cohorts
eFigure 3. Multivariate Analysis of Hazard Ratio for OS in AR Cohorts
eTable 2. Characteristics of Patients With Acquired Resistance

This supplemental material has been provided by the authors to give readers additional information about their work.

© 2022 Zhuo N et al. *JAMA Network Open.*

eFigure 1. Overall survival of initial response patients and acquired resistance patients.

(A) K-M OS analysis of patients with initial response to ICIs (N=373). The median OS for initial response patients was not reached (95% CI, 13.9 to 15.8). (B) K-M OS analysis of patients with AR to ICIs (N=141). The median OS for patients with AR was 20.0 months (95% CI, 13.5 to 16.3).

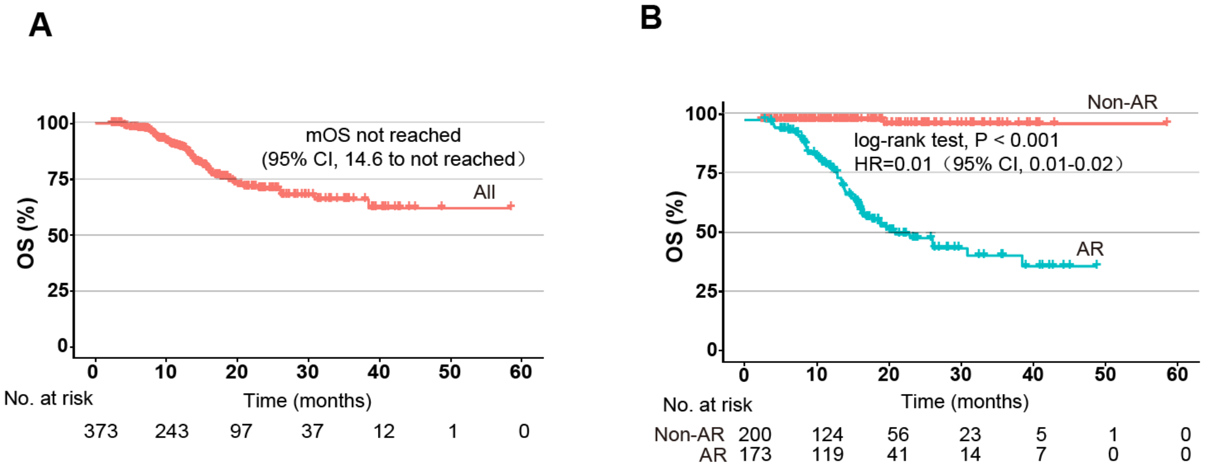

eFigure 2. The rate of acquired resistance over time in different primary sites.

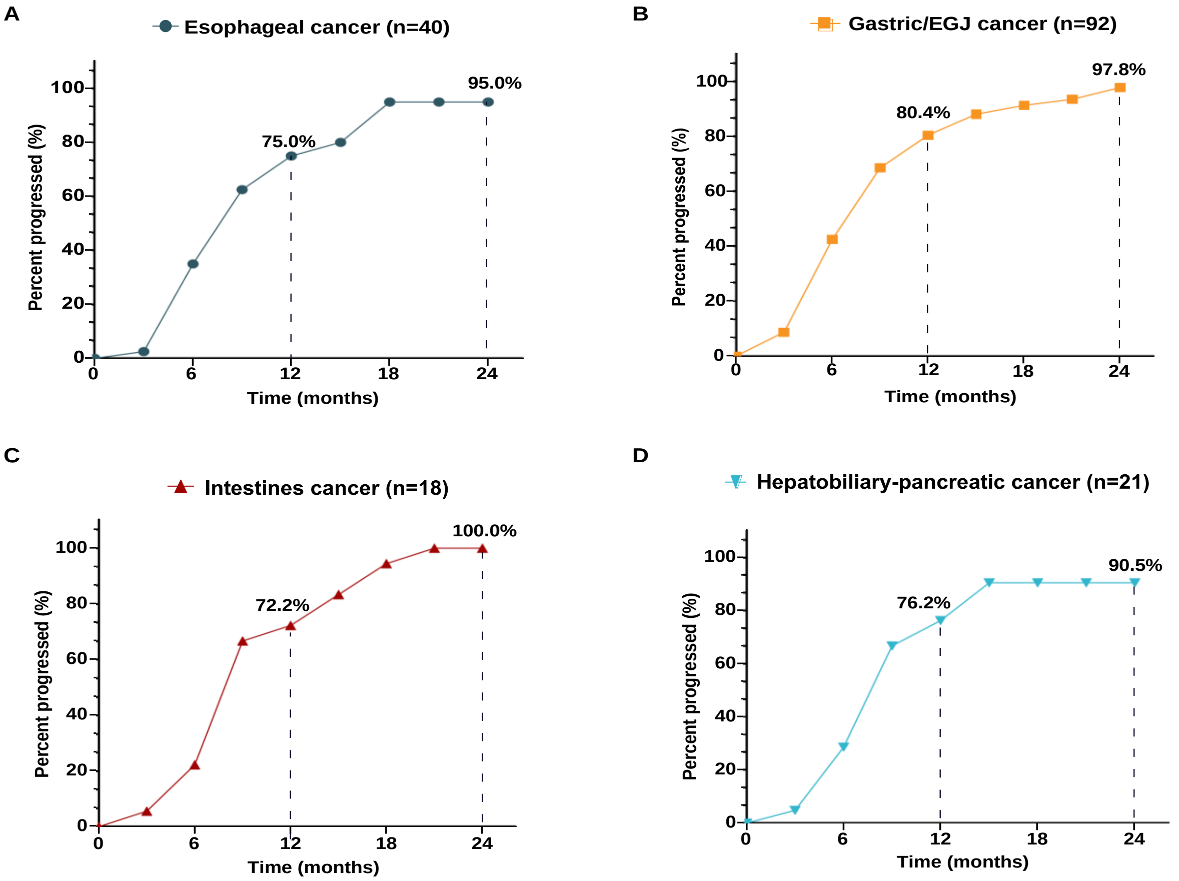

(A-D) The rate of AR over time in patients with esophageal cancer (A)、gastric cancer/EGJ cancer (B)、intestinal cancer (C) and hepatobiliary-pancreatic cancer (D). EGJ, esophagogastric junction.

© 2022 Zhuo N et al. *JAMA Network Open.*

eTable 1. Univariate analysis of hazard ratio for OS in AR cohorts.

Univariate analysis of hazard ratio for OS					
Factors			HR	95% CI	P-value
Age					
	<60	reference			
	≥60		1.1	0.71 - 1.9	0.57
Sex					
	female	reference			
	male		1.8	0.86 - 3.8	0.12
ECOG					
	<2	reference			
	≥2		2	0.95 - 4.2	0.06
Alcohol consumption					
	No	reference			
	Yes		1.2	0.74 - 1.9	0.48
Smoking history					
	No	reference			
	Yes		0.96	0.6 - 1.6	0.88
Previous radiotherapy					
	No	reference			
	Yes		1.1	0.66 - 1.9	0.67
Previous surgery					
	No	reference			
	Yes		0.66	0.34 - 1.3	0.23
Primary tumor site					
	Gastric/EGJ	reference			
	Esophageal		1.33	0.77 - 2.3	0.31
	Intestinal		0.83	0.3 - 2.4	0.73
	Pancreatic		0.44	0.17 - 1.1	0.08
	Hepatobiliary		0.67	0.20 - 2.2	0.5
Histology					
	Adenocarcinoma	reference			
	Squamous		1.4	0.82 - 2.4	0.22
	NET		0.63	0.20 - 2.0	0.45
	Others		0.39	0.05 - 2.8	0.35
Histologic grade					
	High	reference			
	Low		0.51	0.12 - 2.2	0.37
	Medium		0.51	0.12 - 2.1	0.35
Previous systemic therapies					
	1L	reference			
	2L		0.54	0.28 - 1.0	0.06
	3L		0.89	0.51 - 1.6	0.7

Continue

© 2022 Zhuo N et al. *JAMA Network Open.*

Factors			HR	95% CI	P-value
Treatment					
	Combination	reference			
	Mono-ICI		0.85	0.52 - 1.4	0.5
MSI status					
	MSS / pMMR	reference			
	MSI-H / dMMR		0.69	0.35 - 1.3	0.27
Progression pattern					
	Polymetastatic progression	reference			
	Oligoprogression		0.34	0.19 - 0.6	＜0.001

© 2022 Zhuo N et al. *JAMA Network Open.*

eFigure 3. Multivariate analysis of hazard ratio for OS in AR cohorts.

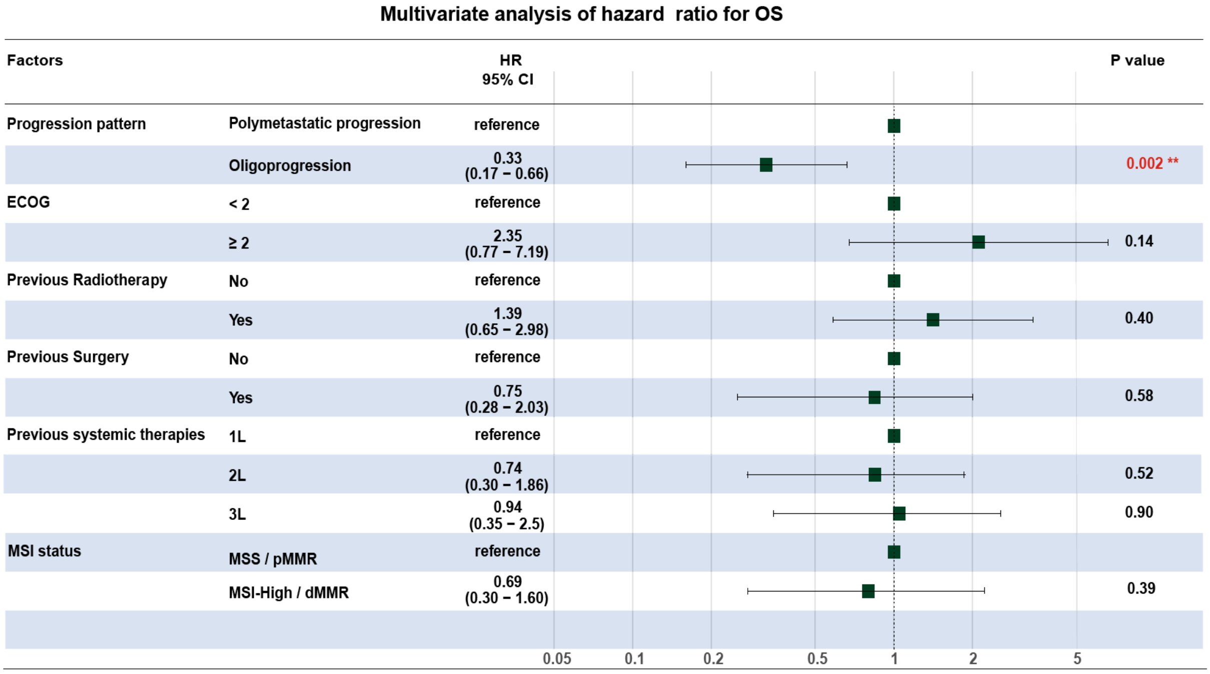

© 2022 Zhuo N et al. *JAMA Network Open.*

eTable 2. Characteristics of patients with acquired resistance.

	**Acquired resistance (N=173) No. (%)**
**Model of progression**	
Original progression*	103 (59.5%)
New Lesions	19 (11.0%)
Original progression* & New leisions	38 (22.0%)
Death	13 (7.5%)
**Site of progression**	
Lymph nodes	101 (58.4%)
Liver	44 (25.4%)
Primary tumor	44 (25.4%)
Peritoneum	30 (21.3%)
Lung	20 (11.6%)
Adrenal gland	11 (7.8%)
Bone	8 (4.6%)
Brain	6 (3.5%)
pleura	5 (2.9%)
Anastomosis	4 (2.3%)
Ovary	3 (1.7%)
Spleen	2 (1.2%)
**Management after progression**	
Chemotherapy	41 (23.7%)
Best supportive care	30 (17.3%)
Maintaining immunotherapy	22 (12.7%)
Phase I clinical trials	21 (12.1%)
Radiotherapy	16 (9.2%)
Targeted therapy	12 (6.9%)
Interventional therapy	7 (4.0%)
Unknown	24 (13.9%)

Original progression* was defined as the progression of lesions which were originally involved before immunotherapy.

© 2022 Zhuo N et al. *JAMA Network Open.*
